# Differential lung ventilation via tracheostomy using two endotracheal tubes in an infant: a case report

**DOI:** 10.1186/s13256-017-1417-x

**Published:** 2017-09-08

**Authors:** Demet Demirkol, Yasemin Ataman, Gökhan Gündoğdu

**Affiliations:** 1 Pediatric Intensive Care Unit, Koç University School of Medicine, Maltepe Mahallesi, Davutpasa Cad, 34010 Istanbul, Turkey; 2 Department of Pediatrics, Bezmialem Vakif University School of Medicine, Istanbul, Turkey; 3 Pediatric Surgery Unit, Koç University School of Medicine, Istanbul, Turkey

**Keywords:** Differential lung ventilation, Asymmetric lung disease, Infant, Tracheotomy, Single-lumen tubes

## Abstract

**Background:**

This case report presents differential lung ventilation in an infant. The aim is to define an alternative technique for performing differential lung ventilation in children. To the best of our knowledge, this is the first report of this kind.

**Case presentation:**

A 4.2-kg, 2.5-month-old Asian boy was referred to our facility with refractory hypoxemia and hypercarbia due to asymmetric lung disease with atelectasis of the left lung and hyperinflation of the right lung. He was unresponsive to conventional ventilator strategies; different ventilator settings were required. To perform differential lung ventilation, two separate single-lumen endotracheal tubes were inserted into the main bronchus of each lung by tracheotomy; the tracheal tubes were attached to discrete ventilators. The left lung was ventilated with a lung salvage strategy using high-frequency oscillatory ventilation, and the right lung was ventilated with a lung-protective strategy using pressure-regulated volume control mode. Differential lung ventilation was performed successfully with this technique without complications.

**Conclusions:**

Differential lung ventilation may be a lifesaving procedure in select patients who have asymmetric lung disease. Inserting two single-lumen endotracheal tubes via tracheotomy for differential lung ventilation can be an effective and safe alternative method.

## Background

Differential lung ventilation (DLV) has been described for managing unilateral or asymmetrical lung diseases [1–5]. With DLV, it is possible to ventilate both lungs with different ventilator settings. This rare mechanical ventilation method is used mostly in adult patients, and anecdotal cases in infants have been reported [1, 2]. Our patient was an infant who was treated successfully with DLV via a tracheotomy using two separate single-lumen endotracheal tubes.

## Case presentation

A 4.2-kg, 2.5-month-old Asian boy was referred to our facility because of severe respiratory failure that was refractory to conventional mechanical ventilation strategies. He was born at 31 weeks of gestation weighing 1620 g and hospitalized at a neonatal intensive care unit for 66 days with the diagnoses of respiratory distress syndrome, bronchopulmonary dysplasia, and sepsis. Four days after discharge, the patient had respiratory failure; he was intubated and transferred to a local intensive care unit. On the third day of hospitalization, he was transferred to our pediatric intensive care unit. On physical examination, he was an intubated patient with a respiratory rate (RR) of 77 breaths/minute, pulse rate 160 beats/minute, blood pressure 90/54 mmHg, and oxygen saturation (SpO_2_) 85%. His breath sounds were decreased in the left lung. A venous blood gas investigation revealed pH 7.07, partial pressure of carbon dioxide 153 mmHg, partial pressure of oxygen 33 mmHg, and bicarbonate 23 mEq/L. Chest radiography showed minimal atelectasis of the left lung and hyperinflation of the right lung, and a tracheal tube was positioned 1 cm above the carina. The patient was initially ventilated using conventional pressure control ventilation (PCV) mode with positive end-expiratory pressure (PEEP) of 8 cmH_2_O, peak inspiratory pressure (PIP) 20 cmH_2_O, RR 20 breaths/minute, and inspiratory time (TI) 0.8 second. However, he had progressively worsening hypoxemia and hypercarbia. Therefore, an inhaled surfactant was given, and high-frequency oscillatory ventilation (HFOV) was applied. In the following days, his respiratory status improved. HFOV was stopped, and conventional ventilation using PCV mode was started. Weaning attempts were unsuccessful, and a tracheotomy was performed.

**Fig. 1:**
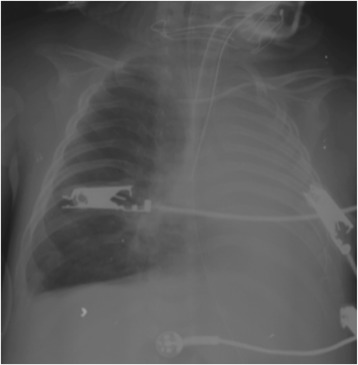
Chest radiographic image showing asymmetric lung injury with severe left-lung atelectasis and right-lung hyperinflation on the 14th day of hospitalization

**Fig. 2:**
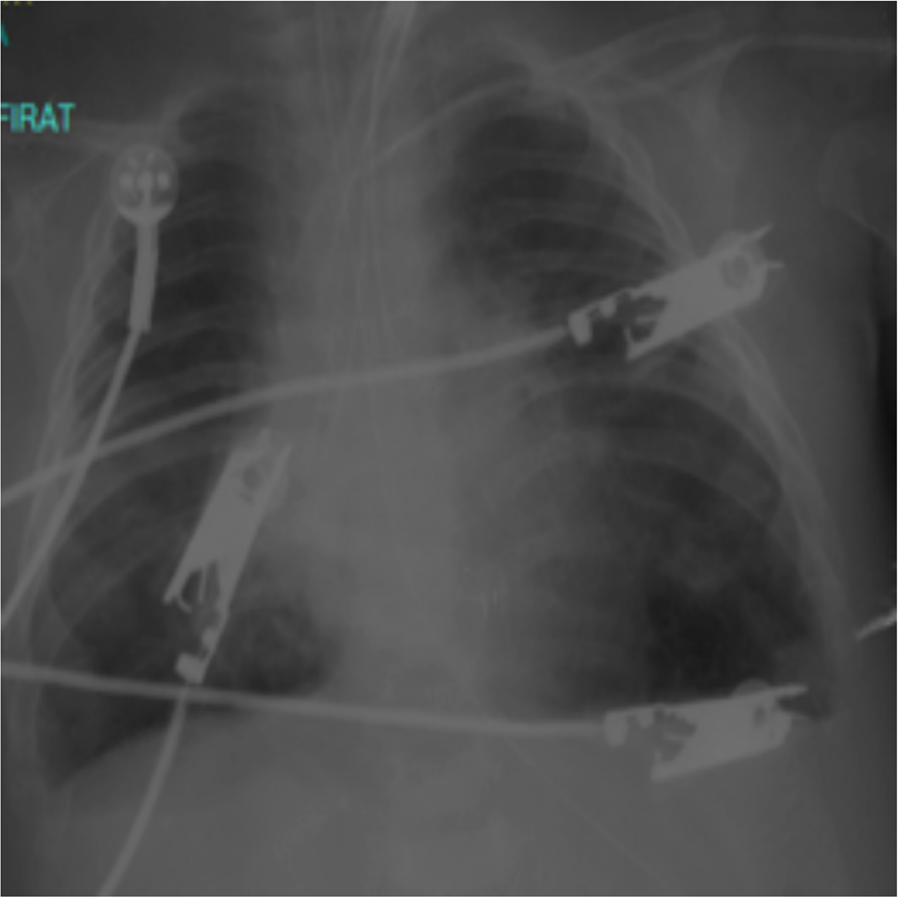
Chest radiographic image revealing that the atelectasis of the left lung and hyperinflation of the right lung were ameliorated after 12 hours of differential lung ventilation

On the 14th day of hospitalization, the patient had a fever and severe hypoxemia with SpO_2_ 40%. A physical examination revealed decreased breath sounds in the left lung. Chest radiography revealed total atelectasis of the left lung and hyperinflation of the right lung (Fig. 1). The mode of ventilation was changed from PCV to pressure-regulated volume control (PRVC), and PEEP and PIP values were increased. The patient’s status did not improve, and his SpO_2_ was 60% with 100% oxygen. A bronchoscopy was performed, and the result was normal. Chest computed tomography revealed hyperinflation and dead spaces in the right lung and total atelectasis of the left lung. Different ventilator strategies were required, so we decided to perform DLV with a lung salvage strategy for the left lung and a lung-protective strategy for the right lung. A 3.0-mm inner-diameter (ID) cuffed tracheal tube and a 2.5-mm ID cuffed tracheal tube were inserted into the left and right bronchi, respectively, via tracheotomy with bronchoscopy in the operating room. These were the largest possible tubes that the main airway diameter would accommodate. The right lung was ventilated with PRVC mode, and ventilator settings were adjusted with PEEP of 5 mmHg, tidal volume 6 ml/kg, RR 15 breaths/minute, and TI 0.6 second. The left lung was ventilated with HFOV, and ventilator settings were adjusted as mean airway pressure of 20 cmH_2_O, amplitude 47 cmH_2_O, frequency 7 Hz, and TI 33%. In the following hours, there was marked improvement, and SpO_2_ was 87% with 75% oxygen. Chest radiography revealed that the atelectasis of the left lung and hyperinflation of the right lung were ameliorated after 12 hours of DLV (Fig. 2). DLV was maintained for 5 days, and ventilator support was reduced in stepwise fashion (Table 1). On the 21st day of hospitalization, the two separate tubing systems were changed to a single-lumen 4.5-mm ID cuffed tracheotomy tube, and conventional ventilation with PRVC mode was started with PEEP of 8 cmH_2_O, PIP 25 cmH_2_O, RR 28 breaths/minute, and TI 0.65 second. Because repeated weaning attempts were unsuccessful, the patient was switched to a home ventilator on the 48th day and was discharged on the 64th day.

**Table 1:** Ventilator settings and blood gas analysis results

		Day of DLV				
		First day	Second day	Third day	Fourth day	Fifth day
Right lung	Mode of ventilation	PRVC	PRVC	PRVC	PRVC	PRVC
	PEEP, cmH_2_O	5	6	6	5	5
	V_T_, ml/kg	6	7	7.5	6.5	6
	Rate, breaths/minute	15	15	15	15	15
	TI, seconds	0.6	0.6	0.6	0.6	0.6
	FiO_2_, %	100	75	65	65	60
Left lung	Mode of ventilation	HFOV	HFOV	HFOV	HFOV	HFOV
	MAP, cmH_2_O	20	23.5	22	20	17.5
	Amplitude, cmH_2_O	47	51	53	48	42
	Frequency, Hz	7	7	7	7	7
	TI, %	33	33	33	33	33
	FiO_2_, %	100	75	65	65	60
Blood gas analysis	pH	7.15	7.22	7.27	7.45	7.42
	pCO_2_, mmHg	81.8	70.2	67.2	58.6	51.8
	pO_2_, mmHg	28.4	42.8	58.2	44.6	56.6
	HCO_3_^−^, mEq/L	28.2	31.7	32.8	38.6	35.5
	BD or BE	−1.5	3.5	8.2	7.9	6.5
	SpO_2_, %	65	87	90	92	96

*Abbreviations: BD* Base deficit, *BE* Base excess, *cmH*_*2*_ *O* centimeter of water, *FiO*_*2*_ Fraction of inspired oxygen, *HCO*_*3*_^−^ Bicarbonate, *HFOV* High-frequency oscillatory ventilation, *Hz* hertz, *MAP* Mean airway pressure, *mmHg* millimeter of mercury, *PEEP* Positive end-expiratory pressure, *pCO*_*2*_ Partial pressure of carbon dioxide, *pH* potential of hydrogen, *pO*_*2*_ Partial pressure of oxygen, *PIP* Positive inspiratory pressure, *PRVC* Pressure-regulated volume control, *SpO*_*2*_ saturation of oxygen, *TI* Inspiratory time, *V*_*T*_ Tidal volume

## Discussion

In this case report, we describe DLV in an infant. DLV was performed by using two separate single-lumen endotracheal tubes, which were inserted into the main bronchus of each lung by tracheotomy. The tracheal tubes were attached to discrete ventilators. To the best of our knowledge, this is the first report demonstrating DLV using two separate endotracheal tubes via tracheotomy in a pediatric patient.

DLV is a rare ventilation technique for patients with severe unilateral or asymmetric lung diseases for whom conventional mechanical ventilation strategies have failed. Causes of unilateral or asymmetric lung diseases include emphysema, pneumonia, trauma, bronchial fistula, atelectasis, aspiration, bronchial artery hemorrhage, or pulmonary edema [1–5]. In these diseases, there are differences in airway resistance, blood flow, and lung and vascular compliance between the two lungs [6]. Airway resistance increases and lung compliance decreases because of the severe inflammatory response in the affected lung. During mechanical ventilation, the gas flow delivered by the mechanical ventilator is directed mostly toward the less affected lung, resulting in overinflation, dead spaces, and parenchymal damage, whereas the afflicted lung is hypoventilated with increased alveolar collapse and decreased regional compliance. Hypoxia may be exacerbated by regional vasoconstriction, and shunting occurs in the capillaries of the affected lung. Therefore, the blood with less oxygen may be ejected into the systemic circulation, and this leads to severe systemic hypoxemia [6]. Alveoli of the less affected lung may be overdistended and may compress the capillaries adjacent to distended alveoli. This leads to increased ventilation-perfusion mismatching and aggravates systemic hypoxemia. Also, right ventricular cardiac work increases and pulmonary blood flow decreases because of higher pulmonary vascular resistance of the overly distended less affected lung [7]. In cases of unilateral or asymmetric lung diseases, conventional bilateral mechanical ventilation strategies may further deteriorate ventilation-perfusion mismatching, worsening hypoxemia and hypercarbia. To provide adequate oxygenation and ventilation, ventilator modes and parameters should be selected according to the different characteristics of each lung, such as compliance, resistance, and blood flow. DLV allows for different ventilator settings to be used for each lung.

DLV was described for the first time in 1931 as a technique in thoracic anesthesia, and its use outside the operating room was first reported in the 1970s. Since then, DLV has been used in the critical care setting [8, 9]. A variety of techniques for performing DLV have been described. The main method in adult patients is to use double-lumen endotracheal or tracheotomy tubes [9, 10]. Although DLV is usually applied with double-lumen tubes, serious complications such as tube displacement, tracheal or bronchial injury, pneumothorax, and emphysema have been described with this method [11, 12]. Pediatric double-lumen tubes are suitable for patients older than 1 year of age. In small children and infants, DLV must be performed by using alternative techniques such as a combination of a laryngeal mask airway (LMA) and a long tracheal tube or two single-lumen tracheal tubes [1, 2, 13].

DLV is rarely used in pediatric patients, and therefore reports of experiences with DLV in infants are limited in the published literature. Arai and Yamashita [2] reported a case of DLV in a 2-month-old infant with unilateral lobar emphysema after repair of a congenital diaphragmatic hernia. The atelectatic right lower lobe was ventilated with HFOV, and the left lung was ventilated via LMA with a conventional ventilator using pressure cycle mode [2]. Hirata *et al*. reported use of DLV in a 5-month-old infant with complete atelectasis of the right lung due to respiratory syncytial virus infection [1]. In these anecdotal cases, a combination of an LMA and a long pediatric endotracheal tube was used to perform DLV. A swivel connector was attached to the LMA, and the tracheal tube was placed into a main bronchus through the LMA via the swivel connector under fluoroscopic guidance. The LMA and the tracheal tube were attached to two different ventilators, and two lungs were ventilated with different ventilator settings [1, 2]. In our patient, we used an alternative technique for performing DLV. With this technique, two single-lumen cuffed endotracheal tubes were inserted into the main bronchus of each lung via tracheotomy under bronchoscopic guidance. We used the cuffed tubes because we planned to inflate the cuffs if there was a leak. However, we did not observe any leak during ventilation. Each tube was placed 1–1.5 cm down the main stem bronchus. The tracheal tubes were attached to different ventilators, each providing support to a lung. Ventilator settings were chosen in accordance with the clinical, laboratory, and radiological results, considering different characteristics of each lung. No complications such as tube displacement, tube obstruction, pneumothorax, emphysema, or bronchial injury occurred. This technique proved to be efficient and simple, and use of this technique with pediatric patients has not previously been reported, to the best of our knowledge. Yamakawa *et al*. reported five adult cases (two with unilateral pneumonia and three with thoracic trauma) managed with DLV by using two single-lumen tubes via tracheotomy without complications, similar to in our patient’s case [14].

## Conclusions

Unilateral and asymmetric lung disease, especially in infants and small children, poses difficult ventilation management problems for intensive care physicians. Considering these problems, DLV may be the correct and convenient ventilation method for patients who need discrete ventilation strategies. Furthermore, we conclude that DLV may be performed effectively and safely in infants and young children via tracheotomy with separate single-lumen endotracheal tubes.

## References

[1] Hirata N, Sato H, Toyoshima Y, Kawana S, Namiki A. Differential lung ventilation by use of a combination of a laryngeal mask airway and an endotracheal tube in a pediatric patient with atelectasis of the right lung. Masui. 2003;52:906–8. 13677291

[2] Arai T, Yamashita M. Differential lung ventilation in an infant using LMA and a long tracheal tube. Paediatr Anaesth. 2003;13:438–40. 10.1046/j.1460-9592.2003.01060.x 12791119

[3] Garlick J, Maxson T, Imamura M, Green J, Prodhan P. Differential lung ventilation and venovenous extracorporeal oxygenation for traumatic bronchopleural fistula. Ann Thorac Surg. 2013;96:1859–60. 10.1016/j.athoracsur.2013.01.094 24182475

[4] Achar S, Chaudhuri S, Krishna HM, Sagar MS. Re-expansion pulmonary oedema- differential lung ventilation comes to rescue. Indian J Anaesth. 2014;58:330–3. 10.4103/0019-5049.135051 PMC4091004 25024481

[5] Cinnella G, Dambrosio CG, Brienza N, Giuliani R, Bruno F, Flore T, Brienza A. Independent lung ventilation in patients with unilateral pulmonary contusion. Intensive Care Med. 2001;27:1860–7. 10.1007/s00134-001-1149-6 11797020

[6] Powner DJ. Differential lung ventilation during adult donor care. Prog Transplant. 2010;3:262–7. 10.1177/152692481002000310 20929111

[7] Yusim Y, Berkenstadt H, Keidan I. Malignant hyperinflation of the nondependent lung during chest surgery. Eur J Anaesthesiol. 2001;18:774–7. 10.1046/j.1365-2346.2001.00923.x 11580787

[8] Tuxen DV. Independent lung ventilation. In: Tobin MJ, editor. Principles and practice of mechanical ventilation. 3rd ed. New York: McGraw-Hill; 2013. p. 629–54.

[9] Glass DD, Tonnesen AS, Gabel JC, Arens JF. Therapy of unilateral pulmonary insufficiency with double lumen endotracheal tube. Crit Care Med. 1976;4:323–6. 10.1097/00003246-197611000-00010 803089

[10] Alberti A, Valenti S, Gallo F, Vincenti E. Differential lung ventilation with a double lumen tracheostomy tube in unilateral refractory atelectasis. Intensive Care Med. 1992;18:479–84. 10.1007/BF01708585 1289373

[11] Sivalingam P, Tio R. Tension pneumothorax, pneumomediastinum, pneumoperitoneum, and subcutaneous emphysema in a 15-year-old Chinese girl after a double lumen tube intubation and one-lung ventilation. J Cardiothorac Vasc Anesth. 1999;13:312–5. 10.1016/s1053-0770(99)90270-7 10392684

[12] Hannallah M, Gomes M. Bronchial rupture associated with the use of double lumen tube in a small adult. Anesthesiology. 1989;71:457–9. 10.1097/00000542-198909000-00027 2774277

[13] Tsujimoto S, Fujiwara S, Tashiro C. How to perform differential lung ventilation in pediatric cases? Anesthesiology. 1999;91:327. 10.1097/00000542-199907000-00060 10422969

[14] Yamakawa K, Nakamori Y, Fujimi S, Ogura H, Kuwagata Y, Shimazu T. A novel technique of differential lung ventilation in the critical care setting. BMC Res Notes. 2011;4:134–8. 10.1186/1756-0500-4-134 PMC3101656 21545715

